# Usability assessment of an electronic health record in a comprehensive dental clinic

**DOI:** 10.1186/2193-1801-2-220

**Published:** 2013-05-12

**Authors:** Siriwan Suebnukarn, Pawornwan Rittipakorn, Budsara Thongyoi, Kwanwong Boonpitak, Mansuang Wongsapai, Panu Pakdeesan

**Affiliations:** Faculty of Dentistry, Thammasat University, Pathumthani, Thailand

**Keywords:** Electronic oral health record, Cognitive ergonomics, Cognitive task analysis, Usability study

## Abstract

In this paper we present the development and usability of an electronic health record (EHR) system in a comprehensive dental clinic.The graphic user interface of the system was designed to consider the concept of cognitive ergonomics.The cognitive task analysis was used to evaluate the user interface of the EHR by identifying all sub-tasks and classifying them into mental or physical operators, and to predict task execution time required to perform the given task. We randomly selected 30 cases that had oral examinations for routine clinical care in a comprehensive dental clinic. The results were based on the analysis of 4 prototypical tasks performed by ten EHR users. The results showed that on average a user needed to go through 27 steps to complete all tasks for one case. To perform all 4 tasks of 30 cases, they spent about 91 min (independent of system response time) for data entry, of which 51.8 min were spent on more effortful mental operators. In conclusion, the user interface can be improved by reducing the percentage of mental effort required for the tasks.

## Introduction

Electronic health record (EHR) is a generic term for integrated, computer-based, health information systems, accessible at the point of care. EHRs were classified on the basis of the International Organization for Standardization (ISO) definition (ISO/DTR 20514 2004). According to this definition, the EHR means a repository of patient data in digital form, stored and exchanged securely, and accessible by multiple authorized users. It contains retrospective, concurrent, and prospective information and its primary purpose is to support continuing, efficient and quality integrated health care. Some of the major advances in EHR use in health care during the past four and a half decades have dealt with relatively mundane matters such as approaches to capturing and storing information, communicating it, retrieving it, and producing and distributing reports (Mantas 2002). These capabilities have greatly reduced transcription errors, improved legibility of reports, eliminated redundancy, facilitated billing and financial functions, and provided a wide variety of other benefits, which indirectly do affect patient safety, health care quality, and efficacy (Poissant et al. 2005;Weiskopf & Weng 2013). Despite their promise, reports of EHR-related safety hazards are now emerging (Singh et al. 2011). Many currently unanswered, legal, ethical, and financial questions threaten the widespread adoption and use of EHRs. Key legal dilemmas that must be addressed in the near-term pertain to the extent of clinicians’ responsibilities for reviewing the entire computer-accessible clinical synopsis from multiple clinicians and institutions, the liabilities posed by overriding clinical decisions support warnings and alerts, and mechanisms for clinicians to publically report potential EHR safety issues (Sittig & Singh 2011).

The main thrust of information technology efforts recently has been to encourage broad adoption of EHRs, and clinical systems and applications based on the EHR that provide various kinds of functionality (Takian et al. 2012). Dentists are increasingly adopting EHRs in their practices (Zvárová et al. 2008;Cederberg & Valenza 2012;Tokede et al. 2013). Adoption rate is high among academic centers, but less so at community hospitals and in office practices. Estimates of adoption vary, depending on what one considers the benefits of an EHR are, ranging from the ability to review laboratory and radiology results or other limited functionality to that of a system that includes computerized physician order entry. Dentists are increasingly adopting EHRs, and are using structured data entry interfaces to enter data such that the data can be easily retrieved and exchanged (Walji et al. 2013). Although some studies have discussed the benefits of EHR in dentistry (Zvárová et al. 2008;Tokede et al. 2013;Hippmann et al. 2010), Poor usability is one of the major barriers against optimum usage of electronic health records (EHRs). It is common for user resistance to challenge implementation efforts. As more dental clinics undertake EHR implementations, a solid understanding of how to foster acceptance of EHR is necessary to reap the benefits of clinical error reduction, improved quality of care, and decreased healthcare costs (Jenkings & Wilson 2007). The principles and practices of human-computer interactions can be used to bolster user satisfaction and increase usability, thereby increasing the chances of being successful in the implementation of EHR (Garrard 2000).

In this paper we present the development and usability of an electronic health record (EHR) system in a comprehensive dental clinic. The graphic user interface of the system has been designed to consider the concept of cognitive ergonomics. The user interface of the EHR was analyzed using the Cognitive Task Analysis (CTA) method called “Goals, Operators, Methods, and Selection rules” (GOMS) (Saitwal et al. 2010) and an associated technique called “Keystroke Level Model” (KLM) (Chi & Chung 1996).The GOMS method was used to evaluate the user interface of the EHR by identifying all sub-tasks of a given task and classifying them into mental (internal) or physical (external) operators. KLM was used to predict task execution time required to perform the given task through the application of its standard set of operators. Our goal is to suggest areas of improvement to promote flexibility in the user interface.

## Materials and methods

### Electronic oral health record system

In this study, we focus on the uses of EHR in a comprehensive dental clinic of final year undergraduates. The dental students take care of the patients in the comprehensive care clinic under supervisions of the faculty members. The dental clinic EHR connects with the hospital EHR. All related medical information, e.g. past medical history, laboratory tests, can be retrieved from the hospital EHR. It integrates specifications of all aspects of oral health care, including history, signs and complaints, prescriptions and procedures for holistic oral health care. The dental section of the system is an open source that can plug in to the hospital EHR. The hospital EHR is the open source program and operates in client–server architecture. The hospital information system is able to connect to the infrastructures and networks of Community hospitals, Province hospitals and health care centers. The data is recorded in a Database Server and used in client/server applications.

Usually, existing electronic oral health records allow the practitioner to electronically document patient care, allow claims transactions to be more quickly and reliably processed as well as being able to communicate with records from other healthcare disciplines. The EHR developed in this study has additional functions. The graphic user interface of each oral health status item has been designed to consider the concept of cognitive ergonomics. Cognitive ergonomics (Hoc 2008), as defined by the International Ergonomics Association, is concerned with mental processes, such as perception, memory, reasoning, and motor response, as these processes affect the interactions among humans and other elements in a system. The graphic user interface of each oral health status item has been designed to follow steps in status recording, from chief complaint to hygiene and periodontal condition, defect and restoration. Graphics for each status item have no ambiguity and are easy to remember.

**Figure 1:**
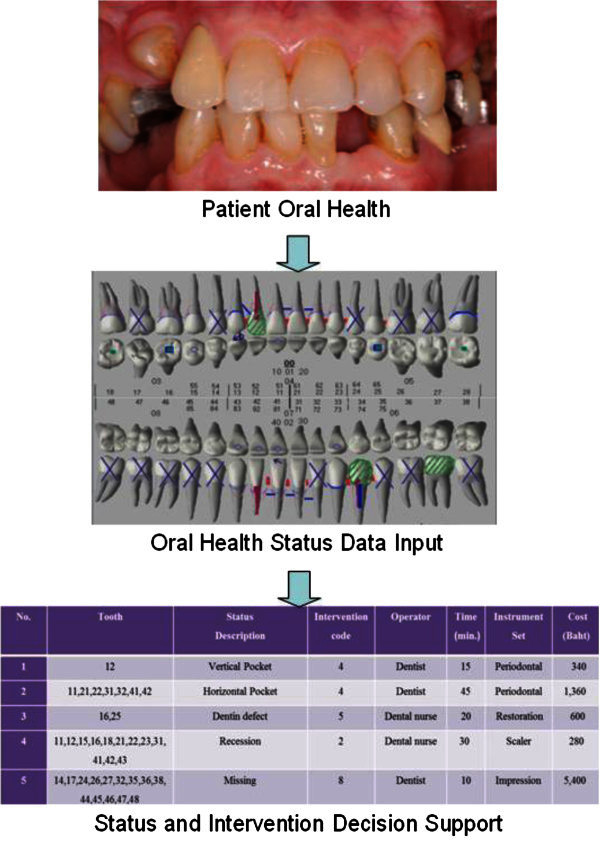
**An example of an oral health status and decision support user interface design after patient’s oral health status recorded in the EHR.**

The status and intervention (SI) index and decision support has been developed to provide the basis for a completely numerical recording system that can cover all of the data on oral status treatment needs, records of planned and completed procedures, clinic organization and scheduling of patients. As for the decision support, the appropriate intervention, care provider, time, setting and cost have been provided for a given oral status which can be altered according to the dentist and patient preferences. The SI index can be used for detailed identification of the treatment needed and which conditions should be referred to the community or province hospitals for treatment. In addition, SI score is an indicator of individual or community health status and of types of intervention in holistic views. SI score can be used for identify the needs of the population to specify the tasks to be accomplished in oral health care, plan sufficient resources for the workplace and community thus allowing for effective performance without unnecessary or excess facilities. The system also enables the epidemiological evaluation of community status and the quantity, quality and effectiveness of care provided; the data can be rapidly and economically summarized, either by hand or by computer. An example of an oral health status user interface is shown in Figure 1. The diagram shows how the program works from users entering data to when they get the decision support message.

### Usability evaluation

To evaluate the user interface of the EHR, we randomly selected 30 paper based cases that had oral examinations for routine clinical care by final year undergraduates. Ten final year undergraduates were recruited and instructed on the use of the EHR and the requirements of the oral health status recording. All participants gave their written informed consent which was approved by the Ethical Review Board of the University. The students had no experience of using EHR and familiarized themselves with the systems interface for 1 week, but they did not familiarize themselves with the task. After the familiarization process, the students recorded the oral health status of the 30 case. Inter-rater reliability was then calculated to determine agreement among students who independently conducted GOMS analysis for each task.

Analyzing a task into Goals, Operators, Methods, and Selection rules is an established method for characterizing a user’s procedural knowledge. When combined with additional theoretical mechanisms, the resulting GOMS model provides a way to quantitatively predict human learning and performance for an interface design, in addition to serving as a useful qualitative description of how the user will use a computer system to perform a task. A GOMS model is a description of the procedural knowledge that a user must have in order to carry out tasks on a device or system; it is a representation of the “how to do it” knowledge that is required by a system in order to get the intended tasks accomplished. Briefly, a GOMS model consists of descriptions of the Methods needed to accomplish specified Goals. The Methods are a series of steps consisting of Operators that the user performs. A Method may call for sub-goals to be accomplished, so the Methods have a hierarchical structure. GOMS is a usability technique that helps to identify lower level perceptual motor issues, quantify the complexity and efficiency of an interface, and evaluate the interface as a whole rather than in isolation. Goals are what the user intends to accomplish (e.g. “locate the patient”). Often goals consist of sub-goals. Operators consist of actions performed to achieve the goal (e.g. “extend hand towards mouse”). Methods are sequences of operators that accomplish a goal (Table 1). Selection rules are used to identify a method in cases where multiple methods may accomplish the same goal.

**Table 1:** **“Locating the patient” task using GOMS along with KLM technique**

Step number	GOMS		KLM	
	Step description	Cognitive distribution Mental/Physical operator	Operators*	Average Time (s) to task completion by each participant
Step 1	Retrieve the name of the patient	Mental	M	1.2
Step 2	Extend the mouse to the patient name	Physical	P	1.1
Step 3	Click on the located patient name	Physical	BB	0.2
Step 4	Extend the mouse to “Oral examination” button	Physical	P	1.1
Step 5	Click on “Oral examination” button	Physical	BB	0.2

*Operators: Point the mouse to target on display (P), Double click (BB), Type a sequence of characters on keyboard (T), Mental operator (M).

GOMS was performed on a set of 4 common tasks that were identified by users of the EHR (Table 2). Two evaluators independently conducted GOMS on each of the 4 tasks. Inter-rater reliability was calculated (using SPSS: Statistical Package for the Social Sciences 16.0) to see the agreement between the total number of steps required to achieve the given task and cognitive distribution (as mental or physical operator) of those tasks.

**Table 2:** **A GOMS analysis for 4 tasks and their subtask**

Task	Task name	Subtask
1	**Locating the patient**	**step 1** Retrieve the name of the patient [M]
		**step 2** Extend the mouse to the patient name [P]
		**step 3** Click on the located patient name [P]
		**step 4** Extend the mouse to “Oral examination” button [P]
		**step 5** Click on“Oral examination” button [P]
**2**	**Enter chief complaint**	**step 1** Think of location in main menu [M]
		**step 2** Extend hand towards mouse and go to chief complaint menu [P]
		**step 3** Click on the chief complaint menu [P]
		**step 4** Retrieve the patient's chief complaint [M]
		**step 5** Type the patient's chief complaint [P]
		**step 6** Extend hand towards mouse and go to save button [P]
		**step 7** Click on save button [P]
		**step 8** Extend hand towards mouse and go to back button [P]
		**step 9** Click on back button and go to main page [P]
**3**	**Document coding of tooth status**	**step 1** Retrieve the data of tooth [M]
		**step 2** Extend hand towards mouse and go to tooth number button/select tooth number [P]
		**step 3** Click on tooth number button [P]
		**step 4** Search for coding of tooth status/ select tooth options [M]
		**step 5** Extend hand towards mouse and go to coding of tooth status button/select tooth status [P]
		**step 6** Click on tooth status [P]
		**step 7** Review coding of tooth status [M]
		**step 8** Click on coding of tooth status [P]
		**step 9** Extend hand towards mouse and go to back button [P]
		**step 10** Click on back button and go to main page [P]
**4**	**Save the data**	**step 1** Review all of tooth status [M]
		**step 2** Extend hand towards mouse and go to save button [P]
		**step 3** Click on save button [P]

Subtask to perform all 4 tasks were identified that comprise of 27 steps in total. Each step is EHR classified into mental [M] or physical [P] operator.

The KLM model was used to estimate the time required to accomplish each of the 4 tasks. The EHR is a standard set of eight operators in KLM with their execution time estimated from experimental data (Saitwal et al. 2010). We did not take into consideration the time taken for the system to respond to the (W(t)) operator, so this analysis is essentially system-independent. In this study, four of those operators are used; Point the mouse to target on display (P), Double click (BB), Type a sequence of characters on keyboard (T) and Mental operator (M). Of these, the first three operators are exclusively physical operators and are estimated to have fixed execution times, except T operator. For T operator, we designed the study to fix all 30 cases to have the same chief complaint. It meant that two evaluators typed the same characters on the keyboard, but may have shown a different duration of execution time. Hence, their total average execution time (T) for a given task can be given by

where*i*= 1, 2, 3, … a ; a is the number of steps on each tasks*t*_*i*_ is the time required to perform *ith* operator, *n*_*i*_ is the number of times *ith*operator is used, and

As far as forth operator – mental operator (M) is concerned, it is estimated that those operators take 1.2 s on average.

## Results

As show in Table 3, the total number of steps for a given task ranged from a minimum of 3 for “Task 4 - Save the data” to a maximum of 10 for “Task3 - Document coding of tooth status”.

**Table 3:** **Summary results of 4 prototypical tasks showing total number of steps, distribution of mental and physical operators, execution times, and inter-rater reliability**

Task No.	TASK name	Total steps	Operator		% Mental	Execution time (s)		Total time (s)	Inter-rater reliability
			Mental	Physical		Mental	Physical		
1	Locating the patient	150	30	120	25	36	67.55	103.55	0.997
2	Enter chief complaint	1020	60	935	6.42	72	340.15	412.15	0.998
3	Document coding of tooth status	6640	2485	4155	59.81	2944	1911.6	4855.6	1
4	Save the data	90	30	60	50	36	33	69	0.998
Average mental operator (%)					35.3075				
Total execution time for all tasks (s)						3088	2352.3	5440.3	
Total execution time for all tasks (%)						56.89	43.11	100	(N=30)

Steps for each of the 4 tasks were further classified as either mental [M] or physical [P] operators depending upon their cognitive distributions based on the GOMS classification. For 30 cases, the results show that of the total operators, 35.30 % of the steps were mental.

The second half of Table 3 estimates the total amount of time the user would take to execute each task in all 30 cases. These values reflect the time a user has spend interacting with the EHR and do not include the time in examining a patient. Further, these estimates are based on the assumption that the users are expert computer users. Execution time for the 4 tasks ranged from a low of 69 s to high of 1911.6 s. The mental operators accounted for about 56.89% of the total time.

Inter-rater reliability was also assessed between the mental/ physical operator classification results by two evaluators (see Table 3). These values range between 0.997 (substantial agreement) and 1 (almost perfect agreement) with the average of 0.9, indicating good reliability of the evaluation method.

**Figure 2:**
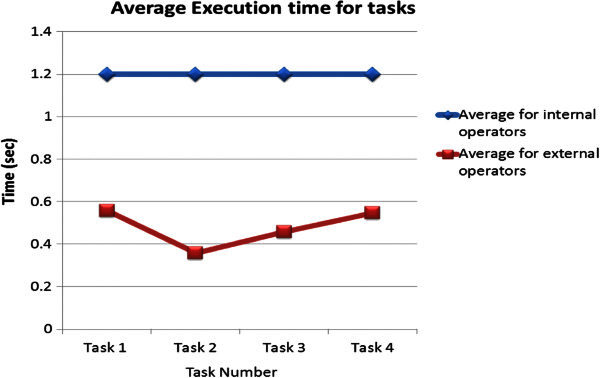
**The average execution times of the participants for all mental (internal) and physical (external) operators for all 4 tasks listed in Table**3**.**

Figure 2 shows the average time required for mental and physical operators in a given task. In particular, the average time for physical steps in each task was calculated for all 4 tasks using Eqs. Note that the average physical operator time vary between 0.36 s and 0.56 s for all tasks because of a different set of clicks and menus in those tasks. Figure 2 also plots the average time for all mental steps, which is always estimated to be 1.2 s. This figure along with the results from Table 3 demonstrates that on average mental steps accounts for 35% of total operators.

## Discussion

EHR systems have the potential to address many limitations of paper-based records and have been described by the Institute of Medicine as an essential technology for medical care (Kassirer 1995).Dental schools have recently begun an ambitious program of converting undergraduate, graduate, and faculty clinics from paper to EHRs (Cederberg & Valenza 2012;Tokede et al. 2013). The functional requirements of EHRs in dental schools are different from those in medicine and nursing. Surveys of students, faculty, and staff before and after implementation indicated that users had mixed feelings about the system in terms of efficiency and time required compared with paper charts. As reported by Walji et al. (2009), the users found that the electronic patient record improved patient care and that they would recommend such a system to dentists starting a new practice. It is essential that the dentists understand the potential benefits of using EHRs in their practices not only for patient care but also for outcome measurements (when linked with other health and social care datasets), quality improvement, public health surveillance, and research.

Researchers in medicine, ergonomics, health economics, cognitive science, and biomedical informatics have conducted several studies on the design of EHRs (Liu et al. 2009;Collins et al. 2011;Cho et al. 2010;Blobel et al. 2003). The general approach that has been used in analyzing the basis for human performance is known as cognitive task analysis (CTA) (Militello & Hutton 1998). Its purpose is to capture the way the mind works―to capture cognition. CTA could describe the basis for skilled performance that is being studied. In using CTA, cognitive scientist try to capture what people are thinking about, what they are paying attention to, the strategies they are using in making decisions, what they are trying to accomplish, what information they discard, and what they know about the way a process works. In studying system usability, there are several possible task analysis techniques that are used to detail the interactions between users and systems. This is done to facilitate and enhanced system design, procedures, training, and support. Task analysis is ideally used when designing a system (Shachak et al. 2009). Using task analysis early in the design process is one way to integrate the study of human processes, including user capabilities and limitations, into the final product. After changes have been made based on task analysis findings, it is important to re-analyze the resulting system to ensure no unforeseen consequences developed as a result of the process. Task analysis could have assisted several authors mentioned in the literature review. The studies by Murff and Kannry (Murff & Kannry 2001) and Payne et al. (2003) investigated various ways that design affects how physicians carry out their work and what physician preferences are. The knowledge gained from these studies is in hindsight. In contrast, a thorough task analysis, by improving the design of a system, can save time and money upfront by reducing the need for revisions. Payne et al. (2003) mentioned that more research in the design stages could have prevented the creation of numerous unnecessary order sets. In this study, we used the cognitive task analysis method called “Goals, Operators, Methods, and Selection rules” and an associated technique called “Keystroke Level Model”. The results suggest that there are many opportunities to improve the efficiency of information delivery and task performance to reduce system complexity. For example, designers could try to simplify the interface and improve the organization to integrate relevant information and data using less windows and screens, or redesign some tasks and functions. Mental workload could be reduced by providing information reminders and recognition-based assistance. For example, some functions like “fill in the blank” could be replaced with “select from a droplist”. Furthermore, we can use colour to classify each main menu on the user interface to reduce mental recall, and also improve the user-friendly interface. For future work, we plan to evaluate the usability of the EHR system on behalf of the user, as in function and representation.

## Conclusions

This study investigated the current user interface of the EHR in the dental student clinic using the cognitive task analysis. The study reveals that the total number of steps required for doing all given tasks. On the other hand, the study also shows that the mental operators are the main part of the total step operators. Further analysis of the execution time shows that more than half of the time is spent in performing mental operators, which can lead to mental fatigue for the users due to extended mental workload for long periods of time. The user interface can be improved by reducing the percentage of mental effort required for the tasks.
